# The Optimization of the Synthesis Process and the Identification of Levobupivacaine Hydrochloride

**DOI:** 10.3390/molecules28227482

**Published:** 2023-11-08

**Authors:** Qiuming Yan, Houjun Gan, Chunzheng Li, Gang Gui, Jianbo Wang, Xiaoming Zha

**Affiliations:** 1 School of Engineering, China Pharmaceutical University, Nanjing 211198, China; 3221081899@stu.cpu.edu.cn (Q.Y.); gerrygui117@163.com (G.G.); 2 China National Medicines Guorui Pharmaceutical Co., Ltd., Huainan 232008, China; 9610073@sina.com; 3 China National Medicines Co., Ltd., Beijing 100077, China

**Keywords:** levobupivacaine hydrochloride, local anesthetic, synthesis, chiral separation, crystal structure, process improvement

## Abstract

In this study, we not only optimized and improved the synthesis process of levobupivacaine hydrochloride (**21**) but also conducted a comprehensive exploration of critical industrial-scale production details, and a novel high-performance liquid chromatography (HPLC) analysis method was developed. Starting with the readily available and cost-effective (*R*,*S*)-*N*-(2,6-dimethylphenyl)piperidine-2-carboxamide (**28**) as the initial material and utilizing l-(–)-dibenzoyl tartaric acid (**29**) for chiral separation, and then through substitution and a salting reaction, levobupivacaine hydrochloride (**21**) was obtained with high purity (chemical purity of 99.90% and enantiomeric excess (*ee*) values of 99.30%). The total yield of the three steps was 45%. Structures of intermediates and the final product were confirmed using nuclear magnetic resonance (NMR) (^1^H NMR, ^13^C NMR), mass spectrometry (MS), and elemental analysis. The crystal structure of the final product was determined through differential scanning calorimetry (DSC), thermogravimetric analysis (TGA), and X-ray diffraction (XRD). Furthermore, we evaluated the risk of the substitution reaction using a reaction calorimeter and accelerating rate calorimetry (ARC). This process offers the advantages of simple operation, greenness, safety, controllable quality, and cost-effectiveness. It provides reliable technical support for the industrial-scale production of levobupivacaine hydrochloride (**21**), which is of significant importance in meeting clinical demands. Pilot-scale production has already been successfully completed by China National Medicines Guorui Pharmaceutical Co., Ltd., with a production scale of 20 kg.

## 1. Introduction

Postoperative pain is observed in almost all postoperative patients, with pain typically concentrated in the first 24–48 h post surgery, and in some cases, it may persist for several days. Failure to manage postoperative pain promptly can easily lead to postoperative complications or the development of chronic pain. Local anesthetics act at the site of administration, with a clear site of action. They can reversibly block the occurrence and transmission of sensory nerve impulses. More importantly, they are convenient to use, simple, require no special examinations for patients, and have a high level of safety with few complications and minimal impact on patients’ physiological functions [1,2]. Levobupivacaine hydrochloride (**21**), chemically known as (2*S*)-1-butyl-*N*-(2,6-dimethylphenyl)-2-piperidinecarboxamide hydrochloride, is an amide-type local anesthetic developed by the UK-based company Cellech Chiroscience. It is the *S*-enantiomer of bupivacaine hydrochloride and is currently marketed in several countries, including the United States, Japan, and China. It is widely used in clinical practice for local anesthesia during surgical and obstetric procedures and postoperative pain management [3,4,5]. Research has shown that the *R*-enantiomer of bupivacaine hydrochloride exhibits significant cardiac toxicity [6]. Therefore, levobupivacaine hydrochloride (**21**), with its lower cardiac toxicity, expands the safety margin for patients.

**Scheme 1:**
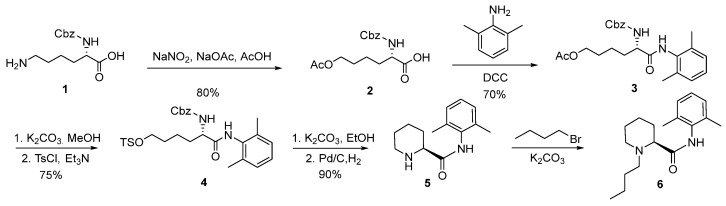
The synthesis of levobupivacaine (**6**) via route 1.

**Scheme 2:**
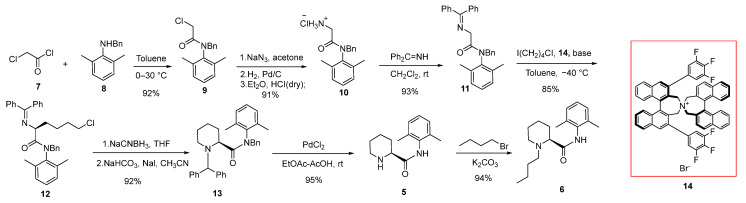
The synthesis of levobupivacaine (**6**) via route 2.

**Scheme 3:**
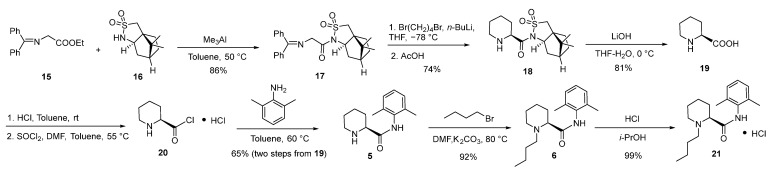
The synthesis of levobupivacaine hydrochloride (**21**) via route 3.

**Scheme 4:**
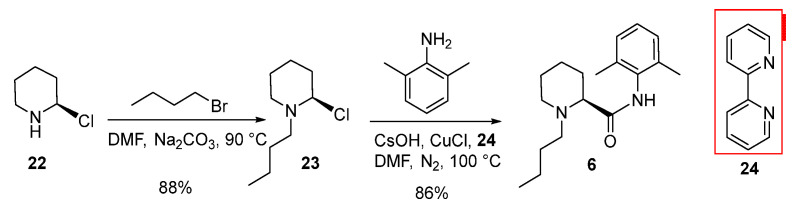
The synthesis of levobupivacaine (**6**) via route 4.

**Scheme 5:**
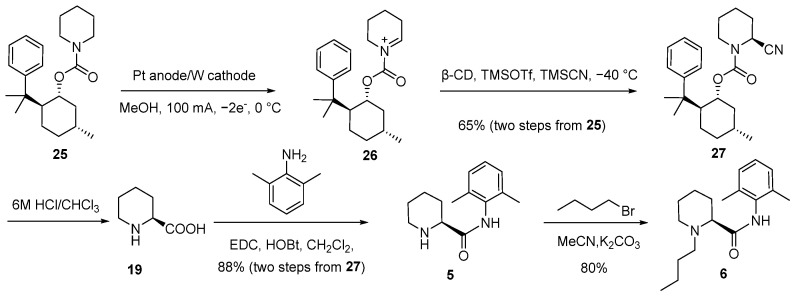
The synthesis of levobupivacaine (**6**) via route 5.

Five main synthetic routes have been reported for the preparation of levobupivacaine (**6**) and its hydrochloride salt, as depicted in Scheme 1, Scheme 2, Scheme 3, Scheme 4 and Scheme 5.

Route 1 begins with *N*-α-Cbz-l-lysine (**1**) as the starting material and involves a five-step process to yield levobupivacaine (**6**) with an *ee* value of 98% and an overall yield of 38% [7]. This route is characterized by its extended series of steps, complex operations, and the requirement for specialized equipment during hydrogenation. Furthermore, dicyclohexylcarbodiimide (DCC) as a condensation agent makes post-processing challenging and unsuitable for large-scale industrial production (Scheme 1).

Route 2 utilizes chloroacetyl chloride (**7**) as the starting material and involves a seven-step process to obtain levobupivacaine (**6**) with an *ee* value of 96% and an overall yield of 54% [8]. This route is characterized by its lengthy reaction sequence, intricate procedures, and the requirement for specialized equipment during hydrogenation. Additionally, the construction of chirality necessitates the use of expensive catalysts (**14**), significantly elevating production costs. Furthermore, the instability and explosive nature of sodium azide pose safety concerns in industrial production (Scheme 2).

Route 3 begins with *N*-(diphenylmethylene)glycine ethyl ester (**15**) as the starting material and involves a seven-step process to yield levobupivacaine hydrochloride (**21**) with an *ee* value of 99% and an overall yield of 31% [9,10]. This route is characterized by its lengthy synthetic steps, where the use of SOCl_2_ as the acylation reagent can lead to equipment corrosion and result in significant contamination. Moreover, the demanding conditions required for chiral synthesis involve costly reagents and complex procedures, making it unsuitable for large-scale industrial production (Scheme 3).

Route 4, utilizing (2*S*)-2-chloropiperidine (**22**) as the starting material, involves a two-step process to obtain levobupivacaine (**6**) with an overall yield of 76% [11]. However, this route faces challenges due to the limited availability of the starting material, (2*S*)-2-chloropiperidine (**22**), from suppliers. Additionally, the product quality is significantly influenced by the quality of the starting material, making it unfavorable for consistent industrial production (Scheme 4).

Route 5 starts with (1*R*,2*S*,5*R*)-5-methyl-2-(1-methyl-1-phenylethyl)cyclohexyl 1-piperidinecarboxylate (**25**) as the starting material. It involves a five-step process to yield levobupivacaine (**6**) with an *ee* value of 91% and an overall yield of 46% [12]. Nevertheless, this route requires specialized equipment, intricate procedures, and stringent requirements for reaction conditions. Furthermore, the starting material is expensive and scarce in supply, and the use of 1-(3-dimethylaminopropyl)-3-ethylcarbodiimide (EDC) for condensation reactions adds to the cost while lowering product quality. In summary, this scheme is not conducive to large-scale industrial production (Scheme 5).

Taking into account the advantages and disadvantages of the five routes for the preparation of levobupivacaine and its hydrochloride salt discussed above, we aim to explore a more feasible approach to facilitate large-scale industrial production. This new method should be capable of overcoming the complexities and high costs associated with existing methods. Consideration should be given to the following factors: (1) Availability and Cost-effectiveness of Starting Materials: The selection of widely available starting materials should be prioritized to reduce production costs while ensuring consistent product quality. (2) Simplified Reaction Steps: We should aim to reduce the number of steps in the synthesis process to make it less complex and to increase the yield of the final product. It is important to avoid using expensive or unstable intermediates and catalysts. (3) Green Synthetic Methods: Preferential consideration should be given to environmentally friendly reaction conditions and reagents to minimize waste generation and emissions of hazardous substances, which contribute to both industrial safety and environmental sustainability. (4) Equipment Accessibility: Ensuring that reaction conditions are applicable to common industrial equipment, without the need for specialized devices, is crucial. (5) Industrial-scale Feasibility: The new route should be economically viable for large-scale industrial production while maintaining high product quality. (6) Optimized Chiral Synthesis: Effective chiral synthesis methods should be employed to ensure high yields and product purity. (7) Safety: The selection of non-explosive reagents and reaction conditions is paramount to ensure industrial safety.

## 2. Results

**Scheme 6:**
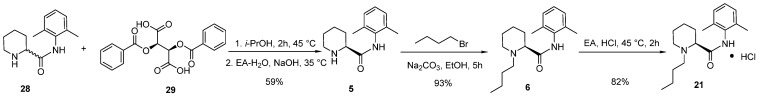
The new synthetic route of levobupivacaine hydrochloride (**21**) (overall yield 45%).

These key factors are crucial in developing a new route for the preparation of levobupivacaine and its hydrochloride salt. Considering these aspects, we optimized the levobupivacaine hydrochloride (**21**) synthesis process (Scheme 6) and developed a novel HPLC analysis method.

### 2.1. Synthesis of (2S)-N-(2,6-Dimethylphenyl)Piperidine-2-Carboxamide (**5**)

In order to obtain a higher purity of (2*S*)-*N*-(2,6-dimethylphenyl)piperidine-2-carboxamide (**5**), we attempted the chiral separation of the readily available and cost-effective (*R*,*S*)-*N*-(2,6-dimethylphenyl)piperidine-2-carboxamide (**28**) following a patented method [13]. During this process, we utilized l-(–)-dibenzoyl tartaric acid (**29**) as the resolving agent and successfully obtained the crude product of (2*S*)-*N*-(2,6-dimethylphenyl)piperidine-2-carboxamide (**5**). Subsequently, based on our refined solvent screening results (Table 1), we ultimately chose to purify it in ethyl acetate (EA) due to higher yields and purity. The overall yield for the resolution and purification was 59% (based on (2*S*)-*N*-(2,6-dimethylphenyl)piperidine-2-carboxamide), with a purity of 99.98%.

### 2.2. Synthesis and Safety Evaluation of Levobupivacaine (**6**)

Intermediate **5** underwent a substitution reaction with bromobutane to obtain (2*S*)-1-butyl-*N*-(2,6-dimethylphenyl)-2-piperidinecarboxamide (Levobupivacaine, **6**). Previous research has found that DMF, as a solvent, presents significant issues for large-scale production, including issues related to impurity formation, unstable yields, and its classification as a high-boiling-point solvent, which may result in residual content in the final product, leading to product nonconformance. Consequently, in selecting reaction conditions, we first considered the solvent type to seek more stable and efficient reaction conditions. To achieve the best results, we conducted extensive experimental research, investigating various reaction conditions, including solvent type, the type of base, reaction time, temperature, and the equivalent amount of bromobutane, to evaluate their impact on reaction yield and purity. Based on our experimental results (Table 2), we ultimately selected **Entry 14** as the reaction condition. Under these reaction conditions, we achieved satisfactory results. The yield reached 93%, indicating the efficiency of the reaction, and the product’s purity reached 99.12%, providing further evidence of high-quality synthesis.

**Figure 1:**
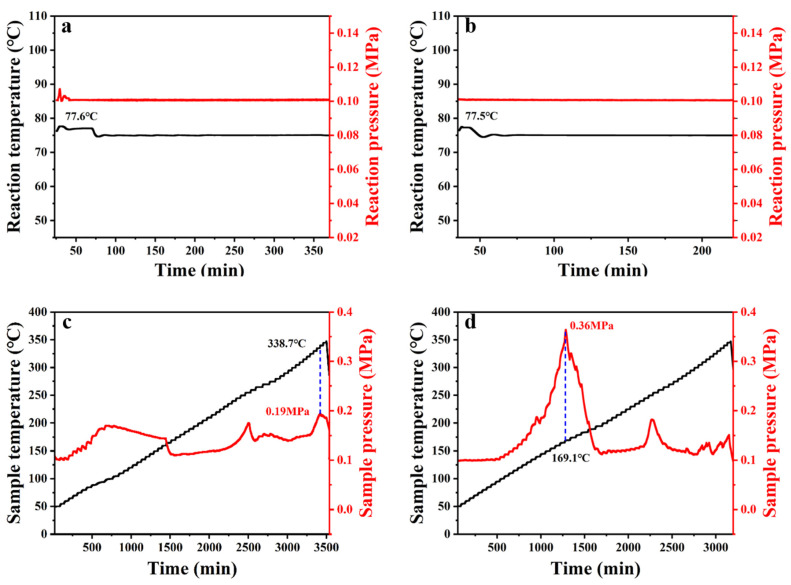
(**a**) Reaction calorimetry of the new reaction; (**b**) reaction calorimetry of the original reaction; (**c**) ARC of the new reaction; (**d**) ARC of the original reaction.

To further investigate the risk level of this reaction in scale-up synthesis, we evaluated the reaction conditions of the new approach (**Entry 14**) and the original approach (**Entry 1**) using the reaction calorimeter and ARC. The purpose of this assessment was to address concerns related to the safety of the new synthesis method when applied on an industrial scale. It is essential to note that small-scale experiments, while seemingly safe, may not adequately represent the conditions and potential hazards associated with industrial-scale production. As shown in Figure 1, using reaction calorimetry, no significant heat release or pressure increase was observed during the reaction under both sets of reaction conditions. Additionally, the maximum reaction temperatures were 77.6 °C and 77.5 °C, respectively, both below the boiling point of the solvent. Although no significant heat release was observed when evaluating both sets of reaction conditions using ARC, there was a noticeable pressure increase (0.36 Mpa) at 169.1 °C under the original reaction conditions. In conclusion, the experimental results presented in Figure 1 hold significant importance in validating the safety and feasibility of the new synthesis method for industrial-scale production. The new approach exhibited higher yields and purity than the original approach and is safer. These results are crucial for the subsequent preparation of levobupivacaine hydrochloride (**21**).

### 2.3. Synthesis and Crystal Structure Determination of Levobupivacaine Hydrochloride (**21**)

We reacted the levobupivacaine (**6**) synthesized in the previous step with hydrochloric acid to obtain crude levobupivacaine hydrochloride (**21**), and we obtained high-quality levobupivacaine hydrochloride (**21**) through recrystallization. Purification is crucial to ensure the high quality of the final product. During this process, we paid particular attention to the choice of recrystallization solvent to achieve the best results. We used various solvents to attempt to overcome potential issues such as impurities, solvent residues, and unstable yields (Table 3). Ultimately, we determined the most suitable recrystallization solvent (*i*-PrOH). By purifying the crude levobupivacaine hydrochloride (**21**) through recrystallization, we successfully obtained high-quality levobupivacaine hydrochloride (**21**) with a yield of 82%. Importantly, it exhibited chemical purity of 99.90% and an *ee* value of 99.30%. This achievement far exceeds the reported *ee* values in the literature (91%~99%) [7,8,9,10,11,12].

**Figure 2:**
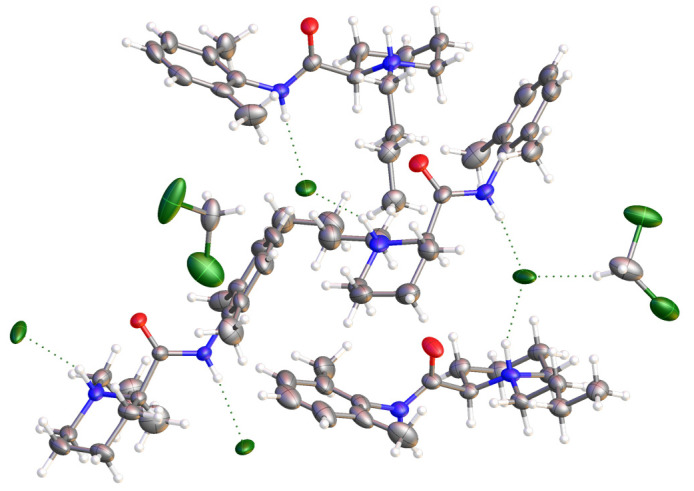
Crystal structure of levobupivacaine hydrochloride (**21**).

**Figure 3:**
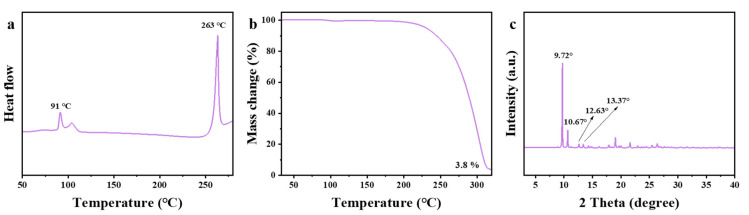
(**a**) DSC of levobupivacaine hydrochloride (**21**); (**b**) TGA of levobupivacaine hydrochloride (**21**); (**c**) XRPD of levobupivacaine hydrochloride (**21**).

Since we prepared a high-quality product, we easily cultivated single crystals of levobupivacaine hydrochloride (**21**) in a mixed solvent of MeOH and DCM (Figure 2 and Table S1), which facilitated the more detailed determination of the spatial structure of the prepared product. To further confirm the crystal form of the product obtained through recrystallization purification, we conducted detailed studies on the final product, levobupivacaine hydrochloride (**21**), using DSC, TGA, and X-ray powder diffraction (XRPD) (Figure 3). The test results consistently indicated that the crystal form of levobupivacaine hydrochloride (**21**) we prepared matched the relatively stable crystal Form A reported in the literature [14]. This further confirms the reliability of our crystal preparation method, which is of significant importance for further research and industrial applications.

Compared to the reported literature, our process requires only three common organic solvents and does not involve column chromatography or other complex operations. The reagents are cost-effective and readily available, and the products are of high quality and low cost, making them suitable for industrial production. Currently, pilot scale-up production has been completed in this enterprise.

## 3. Materials and Methods

### 3.1. General Information

All reagents used were of analytical grade and commercially available. Structural identification was performed using the following instruments: X-4 digital melting point apparatus (Shanghai Precision Scientific Instrument Co., Ltd., Shanghai, China); AV300, AV400, and AV500 nuclear magnetic resonance spectrometers (in D_2_O, CDCl_3_, and DMSO-*d_6_* solvents, Bruker, Billerica, MA, USA); HPLC with Agilent 1260 equipment (Agilent, Santa Clara, CA, USA); liquid chromatography–mass spectrometry (LC-MS) with an Agilent 1260-6230 TOF LC-MS system (Agilent, Santa Clara, CA, USA); elemental analysis with a PerkinElmer 2400 II Organic Elemental Analyzer (PerkinElmer, Waltham, MA, USA); DSC using NETZSCH DSC 3500 (NETZSCH, Selb, Germany); TGA with PerkinElmer TGA 4000 (PerkinElmer, Waltham, MA, USA); XRPD utilizing Rigaku SmartLab SE (Rigaku, Tokyo, Japan); single-crystal X-ray diffraction (SC-XRD) utilizing XtaLAB Synergy-DW (Rigaku, Tokyo, Japan); reaction calorimetry with RC HP-1000A Hangzhou, (Young Instruments, Hangzhou, China); ARC with TAC-500A (Young Instruments, China).

### 3.2. Synthesis

#### 3.2.1. (2*S*)-*N*-(2,6-crystal)Piperidine-2-Carboxamide (**5**)

In a 250 mL reaction flask, (*R*,*S*)-*N*-(2,6-dimethylphenyl)piperidine-2-carboxamide (**28**) (18.0 g, 77.48 mmol), *i*-PrOH (61 mL), and water (36 mL) were added and stirred until dissolved, then heated to 45 °C. A solution of l-(–)-dibenzoyl tartaric acid (**29**) (14 g, 39.07 mmol) in *i*-PrOH (61 mL) was slowly added to the reaction mixture, resulting in the precipitation of a white solid. The mixture was stirred at 45 °C for 2 h and then cooled in an ice bath. After stirring at 0–10 °C for 10 h, the mixture was filtered, and the filter cake was washed with *i*-PrOH to obtain a white solid. The white solid was transferred to a 250 mL reaction flask, and EA (54 mL) and water (54 mL) were added. The pH of the mixture was slowly adjusted to 12.0–14.0 using a 20% NaOH solution at 35 °C, followed by liquid–liquid extraction. The organic phase was successively washed with 0.1 mol/L NaOH solution (75 mL) and water (72 mL). The organic layer was concentrated under reduced pressure to a viscous state, heated to 70 °C, and stirred to dissolve the solid. The mixture was then stirred in an ice bath for 10 h to induce crystallization. After filtration, the solid was washed with cold EA to obtain crude intermediate **5**. Purification: The crude intermediate **5** was transferred to a 100 mL reaction flask, and EA (23 mL) was added. The mixture was heated to 56 °C to dissolve the solid, then cooled in an ice bath. After stirring at 0–10 °C for 12 h, the mixture was filtered, washed with 3 mL of cold EA, and dried under vacuum at 50 °C for 6 h to obtain a white crystalline solid, namely, (2*S*)-*N*-(2,6-dimethylphenyl)piperidine-2-carboxamide (**5**) (5.3 g, 59% (calculated based on (2*S*)-*N*-(2,6-dimethylphenyl)piperidine-2-carboxamide 9 g)), with a purity of 99.98% (HPLC peak area normalization method: Welch C18 column (150 mm × 3.9 mm × 5.0 μm); mobile phase A: MeCN, mobile phase B: 0.02 mol/L phosphate buffer (sodium phosphate buffer, pH = 8.0); 60 min (A:B = 25:75); column temperature 35 °C; flow rate 1.0 mL/min; detection wavelength 210 nm; injection volume 20 µL; sample concentration 0.2 mg/mL; retention time 10.783 min). m.p. 129~130 °C; ESI-MS *m*/*z*: 233.0 [M+H]^+^; ^1^H NMR (CDCl_3_, 300 MHz) *δ* 8.24 (1H, s, CONH), 7.12–7.03 (3H, m, Ar-H), 3.42 (1H, dd, *J* = 9.9, 3.4 Hz, 2-CH), 3.12 (1H, dt, *J* = 12.3, 3.8 Hz, 6-CH), 2.86–2.70 (1H, m, NH), 2.22 (6H, s, Ar-CH_3_), 2.09 (1H, dd, *J* = 12.0, 3.5 Hz, 3-CH), 1.87–1.43 (6H, m, 3-CH, 4-CH_2_, 5-CH_2_, 6-CH); ^13^C NMR (101 MHz, DMSO-*d_6_*) *δ* 172.38, 135.71, 135.52, 128.02, 126.71, 60.26, 45.67, 30.52, 26.35, 24.48, 18.56; Anal. Calcd for C_14_H_20_N_2_O: C, 72.38; H, 8.68; N, 12.06. Found: C, 72.43; H, 8.53; N, 11.86 (Figures S1–S4).

#### 3.2.2. (2*S*)-1-Butyl-*N*-(2,6-Dimethylphenyl)-2-Piperidinecarboxamide (Levobupivacaine, **6**)

In a 100 mL reaction flask, EtOH (26 mL), intermediate **5** (5.31 g, 22.83 mmol), bromobutane (4.50 g, 34.25 mmol), and Na_2_CO_3_ (2.90 g, 27.30 mmol) were added sequentially. The mixture was stirred to disperse and heated to 75 °C. The reaction was carried out for 5 h with TLC monitoring of the reaction. Water (78 mL) was added to the reaction mixture, precipitating a significant amount of light yellow solid. After cooling in an ice bath and stirring at 0–10 °C for 12 h, the mixture was filtered, washed with 56 mL of water, and then subjected to vacuum drying at 50 °C for 6 h. This yielded a light yellow solid, which was (2*S*)-1-butyl-*N*-(2,6-dimethylphenyl)-2-piperidinecarboxamide (Levobupivacaine, **6**) (6.14 g, 93%) with a purity of 99.12% (HPLC peak area normalization method: Welch C18 column (150 mm × 3.9 mm × 5.0 μm); mobile phase A: MeCN, mobile phase B: phosphate buffer (prepared by dissolving 4.9 g of KH_2_PO_4_ and 3.0 g of NaH_2_PO_4_ in water and adjusting the pH to 7.0); 30 min (A:B = 35:65); column temperature 33 °C; flow rate 1.0 mL/min; detection wavelength 210 nm; injection volume 20 µL; sample concentration 1.0 mg/mL; retention time 9.757 min). m.p. 136~137 °C; ESI-MS *m*/*z*: 289.0 [M+H]^+^; ^1^H NMR (CDCl_3_, 400 MHz) *δ* 8.15 (1H, s, CONH), 7.16–7.01 (3H, m, Ar-H), 3.21 (1H, dtd, *J* = 11.8, 3.8, 1.3 Hz, 2-CH), 2.88 (1H, dd, *J* = 10.4, 3.6 Hz, 6-CH), 2.87–2.77 (1H, m, 7-CH), 2.32–2.21 (1H, m, 3-CH), 2.25 (6H, s, Ar-CH_3_), 2.17–2.06 (1H, m, 7-CH, 7-CH), 2.03 (1H, dd, *J* = 11.6, 2.8 Hz, 6-CH), 1.84–1.21 (9H, m, 3-CH, 4-CH_2_, 5-CH_2_, 8-CH_2_, 9-CH_2_), 0.92 (3H, t, *J* = 7.3 Hz, 10-CH_3_); ^13^C NMR (101 MHz, DMSO-*d_6_*) *δ* 172.35, 135.81, 135.59, 128.14, 126.82, 67.99, 56.39, 51.54, 30.65, 28.77, 25.38, 23.60, 20.73, 18.61, 14.45; Anal. Calcd for C_18_H_28_N_2_O: C, 74.96; H, 9.79; N, 9.71. Found: C, 74.48; H, 9.66; N, 9.45 (Figures S5–S8).

#### 3.2.3. Levobupivacaine Hydrochloride (**21**)

In a 100 mL reaction flask, levobupivacaine (**6**) (6.14 g) and EA (31 mL) were added, and the mixture was heated to 45 °C. The solid gradually dissolved, and 2.45 g of hydrochloric acid was slowly added to adjust the pH to 2.5–3.5. The mixture was stirred for an additional 2 h. It was then placed in an ice bath, and stirring was continued for 12 h. The mixture was filtered, and the filter cake was washed with 8 mL of EA. The resulting material was vacuum-dried at 50 °C for 6 h to obtain crude levobupivacaine hydrochloride (**21**). Purification: In a 100 mL reaction flask, the crude product and *i*-PrOH (30 mL) were added, and the mixture was heated to 70 °C with stirring to dissolve the solid. The hot reaction mixture was filtered, and the filtrate was heated again to 70 °C to redissolve any remaining solid. The mixture was then cooled in an ice bath and stirred at 0–10 °C for 12 h. It was filtered, and the filter cake was washed with 5 mL of cold *i*-PrOH. The resulting white solid was levobupivacaine hydrochloride (**21**) (5.63 g, 82%), with a chemical purity of 99.90% (HPLC peak area normalization method: Welch C18 column (150 mm × 3.9 mm × 5.0 μm); mobile phase A: MeCN, mobile phase B: phosphate buffer (prepared by dissolving 4.9 g of KH_2_PO_4_ and 3.0 g of NaH_2_PO_4_ in water to a total volume of 1000 mL, adjusting the pH to 6.9); 40 min (A:B = 33:67); column temperature 33 °C; flow rate 1.0 mL/min; detection wavelength 210 nm; injection volume 20 µL; sample concentration 1.0 mg/mL; retention time 10.423 min), and an *ee* value of 99.30% (HPLC peak area normalization method: DAICE CHIRALPAK IG-3 column (250 mm × 4.6 mm × 3 μm); mobile phase A: MeCN, mobile phase B: phosphate buffer (10 mmol/L KH_2_PO_4_-10 mmol/L NaH_2_PO_4_, mixed in a 1:1 ratio, and the pH was adjusted to 7.0 using phosphoric acid or ammonia); 20 min (A:B = 70:30); column temperature 25 °C; flow rate 0.5 mL/min; detection wavelength 210 nm; injection volume 10 µL; sample concentration 0.5 mg/mL; retention time 15.087 min). m.p. 254~256 °C; ESI-MS *m*/*z*: 289.2 [M-Cl]^+^; ^1^H NMR (Deuterium Oxide, 400 MHz) δ 7.28–7.14 (3H, m, Ar-CH), 4.23–4.07 (1H, m, 2-CH), 3.71 (1H, d, J = 12.5 Hz, 6-CH), 3.14 (3H, tt, J = 13.4, 8.3 Hz, 6-CH, 7-CH_2_), 2.41 (1H, d, J = 14.1 Hz, 3-CH), 2.17 (6H, s, Ar-CH_3_), 1.97 (3H, d, J = 12.1 Hz, 3-CH, 5-CH_2_), 1.87–1.61 (4H, m, 4-CH_2_, 8-CH_2_), 1.36 (2H, h, J = 7.4 Hz, 9-CH_2_), 0.90 (3H, t, J = 7.4 Hz, 10-CH_3_); ^13^C NMR (Deuterium Oxide, 101 MHz) δ 168.37, 135.72, 131.95, 128.56, 128.36, 65.81, 56.15, 52.14, 28.99, 25.14, 22.35, 20.91, 19.35, 17.33, 12.74; Anal. Calcd for C_18_H_29_ClN_2_O: C, 65.51; H, 8.90; N, 8.12. Found: C, 65.33; H, 9.10; N, 8.23 (Figures S9–S13).

### 3.3. Characterization Methods

Reaction calorimetry and ARC were used for the assessment of substitution reaction risks. DSC, TGA, XRPD, and SC-XRD were utilized for the characterization of the final product’s crystalline form and spatial structure. The specific conditions were as follows.

#### 3.3.1. Reaction Calorimeter

The reaction calorimeter was operated under the following conditions: The reaction calorimeter was used to measure real-time heat release rates within the reaction vessel, providing thermal behavior information such as the total heat of the reaction. This allowed for the estimation of the adiabatic temperature rise of the target reaction and the maximum temperature of the runaway system for synthesis reaction (MTSR) that the system can reach in the case of runaway reactions. This information is crucial for assessing the severity of reaction runaways and the process hazard level. The heat flow method was employed with pre- and post-calibration power set at 20W. Following the synthesis process and based on the specified material ratio, approximately 100 g of intermediate **5** was used. Temperature and pressure variations were continuously monitored throughout the entire process.

#### 3.3.2. ARC

The conditions for the ARC experiment were as follows: The thermal runaway characteristics of the substitute reaction were tested through ARC. A stainless steel bomb containing approximately 1.0–1.2 g of the sample was embedded in the equipment. The classic heat–wait–search (H-W-S) mode was adopted in the range of 50–350 °C. The heating interval between each target temperature was 5 °C, and each target temperature was maintained for 30 min to examine the self-exothermic behavior. Throughout the entire process, real-time variations in temperature and pressure were automatically monitored. The adiabatic data obtained from this experiment can be subsequently utilized to calculate critical thermal safety parameters for the assessment of reaction hazards.

#### 3.3.3. DSC

The conditions for the DSC experiment were as follows: The temperature range of the experiment was set from 50 °C to 280 °C, with a heating rate of 10.0 K/min. The DSC instrument used was the DSC 3500 equipped with a t-sensor. The measurement mode and type were set to DSC/sample with a ratio of 1/1. The sample was contained in a Concavus aluminum crucible with a pierced lid. The experiment was conducted in a nitrogen atmosphere with a flow rate of 60.0 mL/min during the heating and 80.0 mL/min during the measurement.

#### 3.3.4. TGA

The conditions for the TGA experiment were as follows: The temperature range of the experiment was set from 30 °C to 320 °C, with a heating rate of 10.0 K/min. The sample was contained in a ceramic crucible. The experiment was conducted in a nitrogen atmosphere with a flow rate of 20.0 mL/min during the heating and measurement.

#### 3.3.5. XRPD

The XRPD instrument was operated under the following conditions: X-ray generation was achieved with a voltage of 40 kV and a current of 50 mA. The scanning mode was 1D, and the primary beam was set to the standard configuration. The scan speed was maintained at 10.00°/min, and data were collected with a counting time of this speed. A standard goniometer with a step width of 0.02° was employed for precise measurements. Additionally, a Cu Kβ filter was utilized to select the X-ray wavelength, and the scan range covered a range from 3 to 40v. Slits, specifically BB (entrance slit box 1/4deg), were chosen to define the beam. The detector employed was the D/teX Ultra 250, and the system incorporated no detector monochromator. Length-limited slits of 10 mm were used, and both receiver slit boxes were kept open during the experiment.

## 4. Conclusions

In this study, starting from the use of cost-effective (*R*,*S*)-*N*-(2,6-dimethylphenyl)piperidine-2-carboxamide, we successfully synthesized high-purity levobupivacaine hydrochloride (**21**) through chiral separation, substitution, and salification reactions, with a total yield of 45%. Additionally, we developed a novel HPLC analysis method to determine the product’s purity, and the results showed chemical purity of 99.90% and an *ee* value of 99.30%. The intermediates and final products were confirmed through ^1^H NMR, ^13^C NMR, MS, and elemental analysis. The final product was also subjected to DSC, TGA, and XRD tests, with results consistent with literature reports. Furthermore, we evaluated the risk of the substitution reaction using a reaction calorimeter and ARC, and the results indicated low risk. The improved process route has the following advantages: (1) a short synthesis pathway and simplified operations without the need for column chromatography, hydrogenation, or other special operations, significantly reducing labor and equipment costs; (2) the entire route avoids expensive catalysts, highly toxic reagents, and high-boiling-point solvents, using only three conventional organic solvents, demonstrating environmental friendliness and high safety while significantly reducing material and waste treatment costs; (3) the final product, levobupivacaine hydrochloride (**21**), shows chemical purity of 99.90% and an *ee* value of 99.30%, and its technical and quality advantages have been validated through pilot-scale production. In summary, this process route offers significant advantages such as simplicity, greenness, safety, quality controllability, and cost-effectiveness, making it highly suitable for industrial production. The process route has already undergone successful pilot-scale production in a commercial setting.

## Figures and Tables

**Table 1:** Optimization of purification conditions in the synthesis of **5**.

Entry	Solvent	Yield (%)	Chemical Purity (%)
1	Acetone	58	98.31
2	EtOH	30	99.99
3	*i*-PrOH	40	99.95
4	4-Methyl-2-pentanone	60	97.53
5	EA	59	99.98

**Table 2:** Substitution reaction condition optimization in the synthesis of **6**.

Entry	Solvent	Base	Bromobutane (eq)	Time (h)	Temperature (°C)	Yield (%)	Chemical Purity (%)
1	DMF	K_2_CO_3_	1.05	3	75	82	87.43
2	EtOH	K_2_CO_3_	1.05	3	75	64	78.60
3	*i*-PrOH	K_2_CO_3_	1.05	3	75	64	72.30
4	MeCN	K_2_CO_3_	1.05	3	75	58	70.05
5	THF	K_2_CO_3_	1.05	3	75	60	73.18
6	EtOH	Na_2_CO_3_	1.05	3	75	66	79.01
7	EtOH	DIPEA	1.05	3	75	49	62.36
8	EtOH	K_3_PO_4_	1.05	3	75	57	75.12
9	EtOH	NaOH	1.05	3	75	25	46.52
10	EtOH	Na_2_CO_3_	1.25	3	75	73	87.29
11	EtOH	Na_2_CO_3_	1.50	3	75	78	91.63
12	EtOH	Na_2_CO_3_	1.75	3	75	80	93.45
13	EtOH	Na_2_CO_3_	2.00	3	75	80	95.59
14	EtOH	Na_2_CO_3_	1.50	5	75	93	99.12
15	EtOH	Na_2_CO_3_	1.50	8	75	93	98.97
16	EtOH	Na_2_CO_3_	1.50	12	75	93	98.85
17	EtOH	Na_2_CO_3_	1.50	5	40	69	83.54
18	EtOH	Na_2_CO_3_	1.50	5	100	89	98.94
19	DMF	Na_2_CO_3_	1.50	5	75	85	85.94

**Table 3:** Optimization of purification conditions in the synthesis of **21**.

Entry	Solvent	Yield (%)	Chemical Purity (%)	*ee* (%)
1	Water	85	98.48	97.12
2	*i*-BuOH	57	99.52	99.12
3	*i*-PrOH	82	99.90	99.30
4	EtOH	73	99.87	98.58

## Data Availability

Data is contained within the article.

## Supplementary Materials

The following supporting information can be downloaded at: https://www.mdpi.com/article/10.3390/molecules28227482/s1. CCDC number 2295308 contains the supplementary crystallographic data for this paper. These data can be obtained free of charge via http://www.ccdc.cam.ac.uk/conts/retrieving.html (or from the CCDC, 12 Union Road, Cambridge CB2 1EZ, UK; Fax: +44-1223-336033; E-mail: deposit@ccdc.cam.ac.uk). Electronic Supplementary Materials (ESM) available: Table S1, Figures S1–S13.
