# Supplementary material for: The Optimization of the Synthesis Process and the Identification of Levobupivacaine Hydrochloride
Source: Molecules. 2023 Nov 8;28(22):7482. doi: 10.3390/molecules28227482 (PMC10673229; doi:10.3390/molecules28227482)
Supplement: Supplementary file 1 [file molecules-28-07482-s001.zip › molecules-2664758-supplementary.pdf]

Supporting Information

The Optimization of the Synthesis Process and the Identification of Levobupivacaine Hydrochloride

Qiuming Yan [1,†], Houjun Gan [2,†], Chunzheng Li [1], Gang Gui [1], Jianbo Wang [3,*] and Xiaoming Zha [1,*]

[1] School of Engineering, China Pharmaceutical University, Nanjing 211198, China; 3221081899@stu.cpu.edu.cn (Q.Y.); gerrygui117@163.com (G.G.)
[2] China National Medicines Guorui Pharmaceutical Co., Ltd., Huainan 232008, China; 9610073@sina.com
[3] China National Medicines Co., Ltd., Beijing 100077, China
[*] Correspondence: xmzha@cpu.edu.cn (X.Z.); 15010153039@163.com (J.W.)
[†] These authors contributed equally to this work.

Table of contents

Table S1. Single crystal X-ray diffraction data of levobupivacaine hydrochloride (21).
Figure S1-S4. NMR spectra, LC-MS spectra, and HPLC of intermediate 5.
Figure S5-S8. NMR spectra, LC-MS spectra, and HPLC of levobupivacaine (6).
Figure S9-S13. NMR spectra, LC-MS spectra, and HPLC of levobupivacaine hydrochloride (21).

Table S1. Single crystal X-ray diffraction data of levobupivacaine hydrochloride (21).

Identification code	levobupivacaine hydrochloride **(1)**
Empirical formula	$C_{37}H_{60}Cl_4N_4O_2$
Formula weight	734.69
Temperature/K	99.99(10)
Crystal system	tetragonal
Space group	$P4_3$
a/Å	15.56180(10)
b/Å	15.56180(10)
c/Å	34.3564(4)
α/°	90
β/°	90
γ/°	90
Volume/Å^3	8320.08(14)
Z	8
ρ_{calc}g/cm^3	1.173
μ/mm^{-1}	2.849
F(000)	3152.0
Crystal size/mm^3	0.42 × 0.23 × 0.13
Radiation	Cu Kα (λ = 1.54184)
2Θ range for data collection/°	5.144 to 134.118
Index ranges	$-18 \leq h \leq 16$, $-18 \leq k \leq 7$, $-40 \leq l \leq 40$
Reflections collected	28449
Independent reflections	13025 [R_{int} = 0.0270, R_{sigma} = 0.0340]
Data/restraints/parameters	13025/1280/860
Goodness-of-fit on F^2	1.035
Final R indexes [I>=2σ (I)]	R_1 = 0.0495, wR_2 = 0.1255
Final R indexes [all data]	R_1 = 0.0512, wR_2 = 0.1269
Largest diff. peak/hole / e Å^{-3}	0.57/-0.54
Flack parameter	0.003(5)

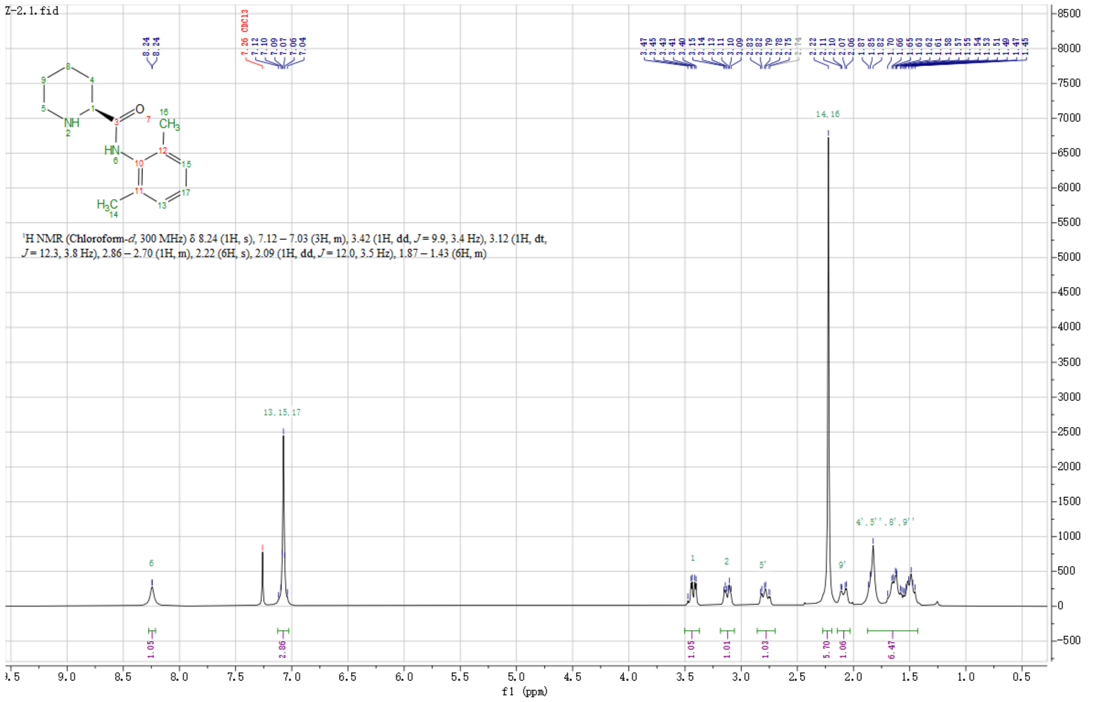

Figure S1 ^{1}H NMR of compound **5** ($CDCl_3$)

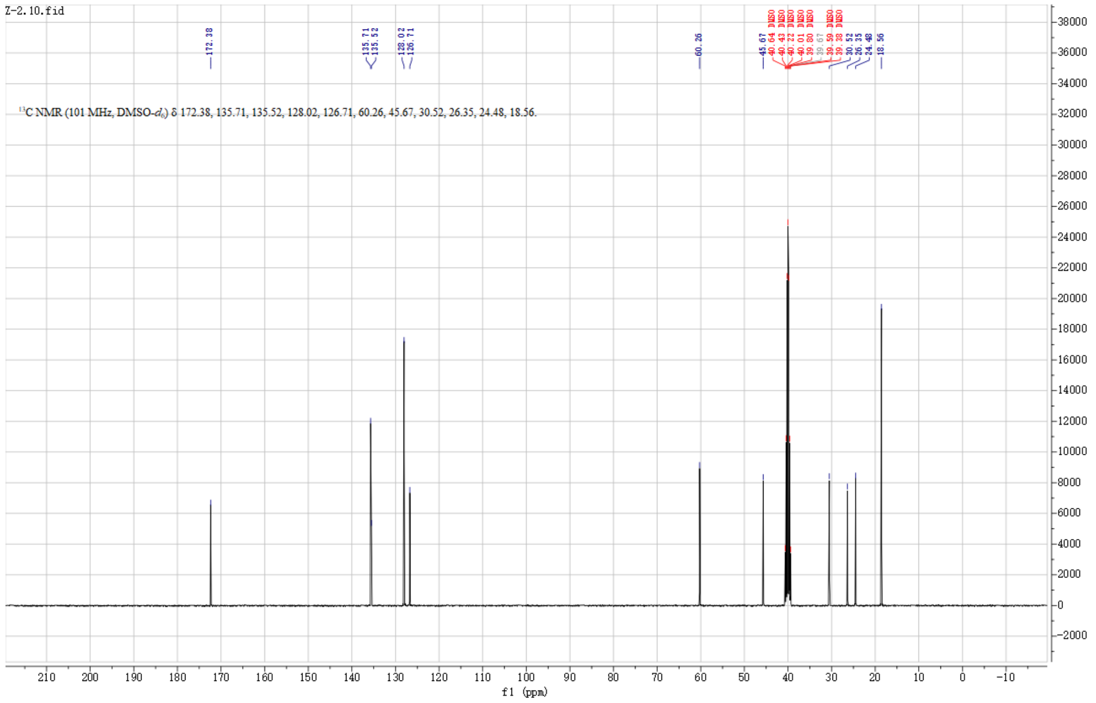

Figure S2 ^{13}C NMR of compound **5** (DMSO-d_6)

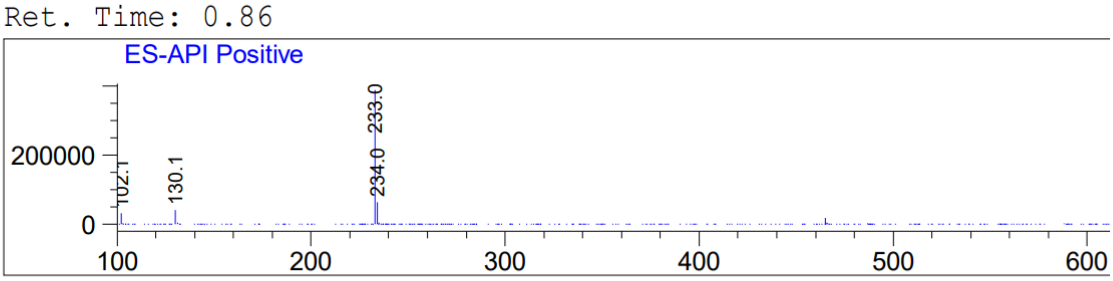

Figure S3 LC-MS of compound **5** (MW =232.2)

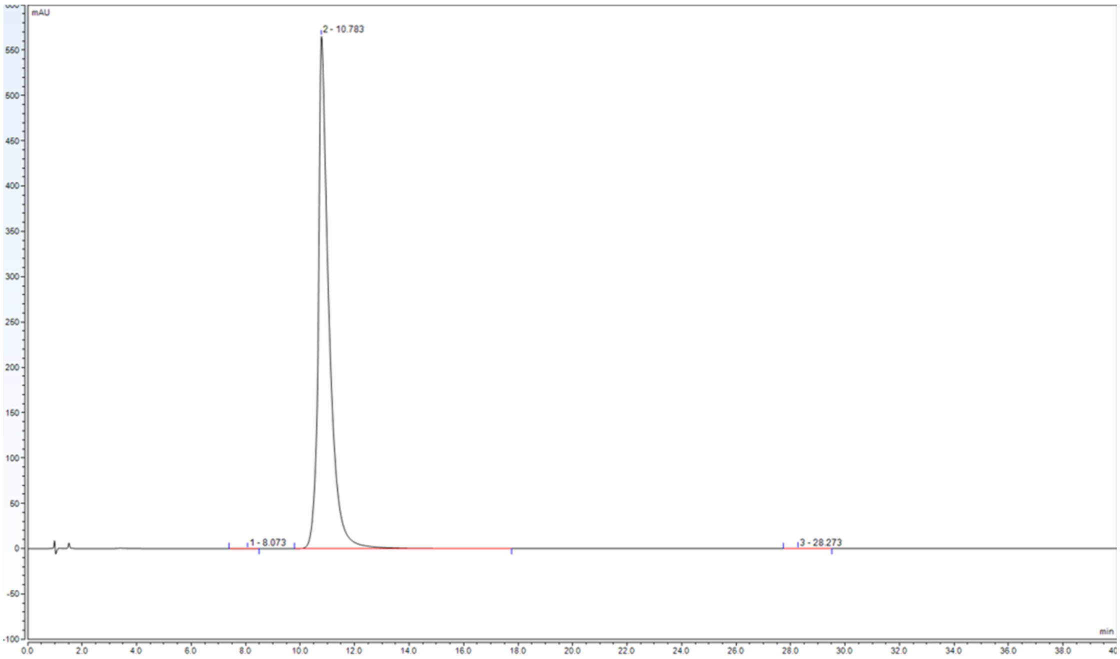

No.	Peak name	Retention Time min	Relative Area %	Area mAU*min
1		8.073	0.01	0.0192
2		10.783	99.98	256.6985
3		28.273	0.01	0.0262

Figure S4 HPLC of compound **5**

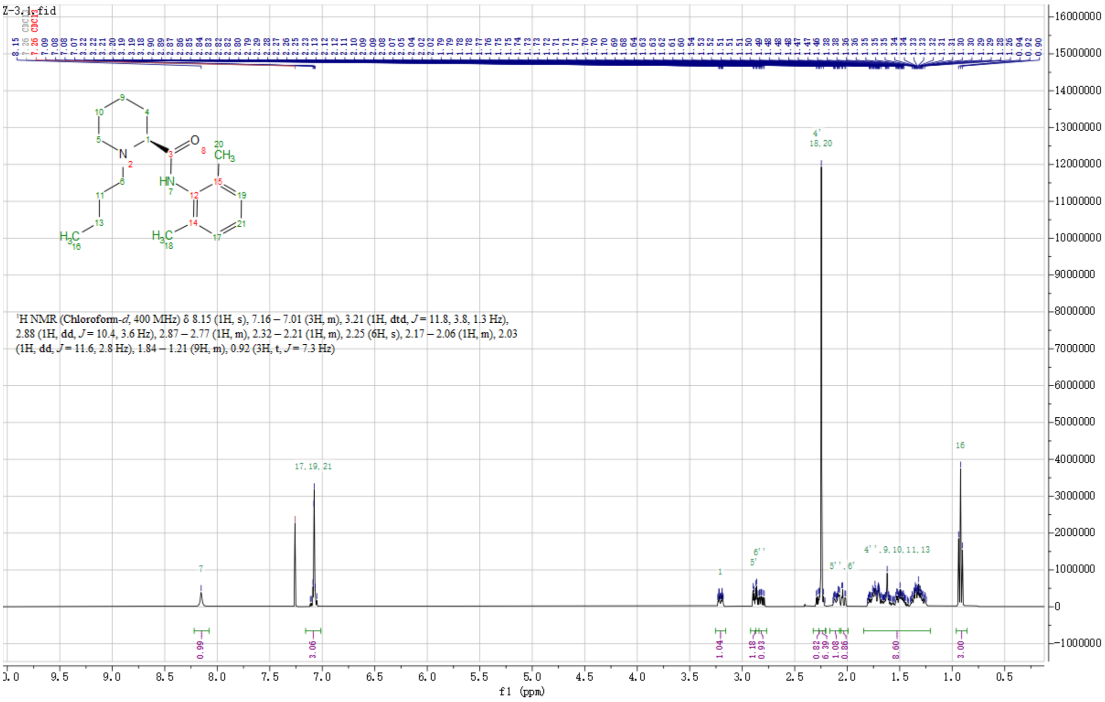

Figure S5 ^{1}H NMR of compound **6** ($CDCl_3$)

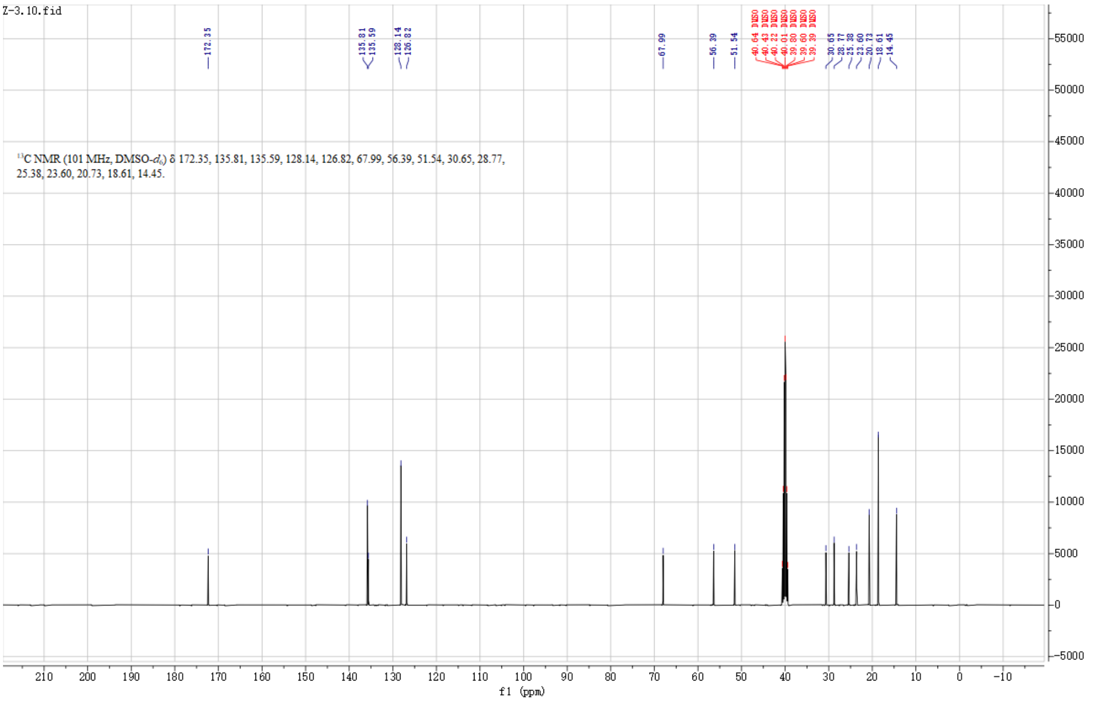

Figure S6 ^{13}C NMR of compound **6** (DMSO-d_6)

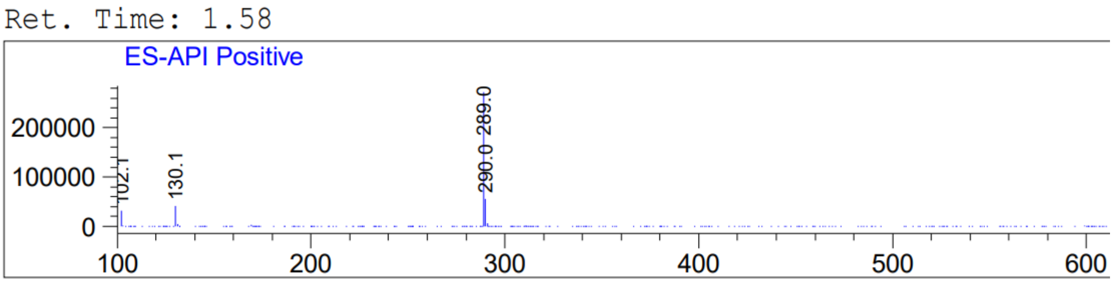

Figure S7 LC-MS of compound **6** (MW =288.2)

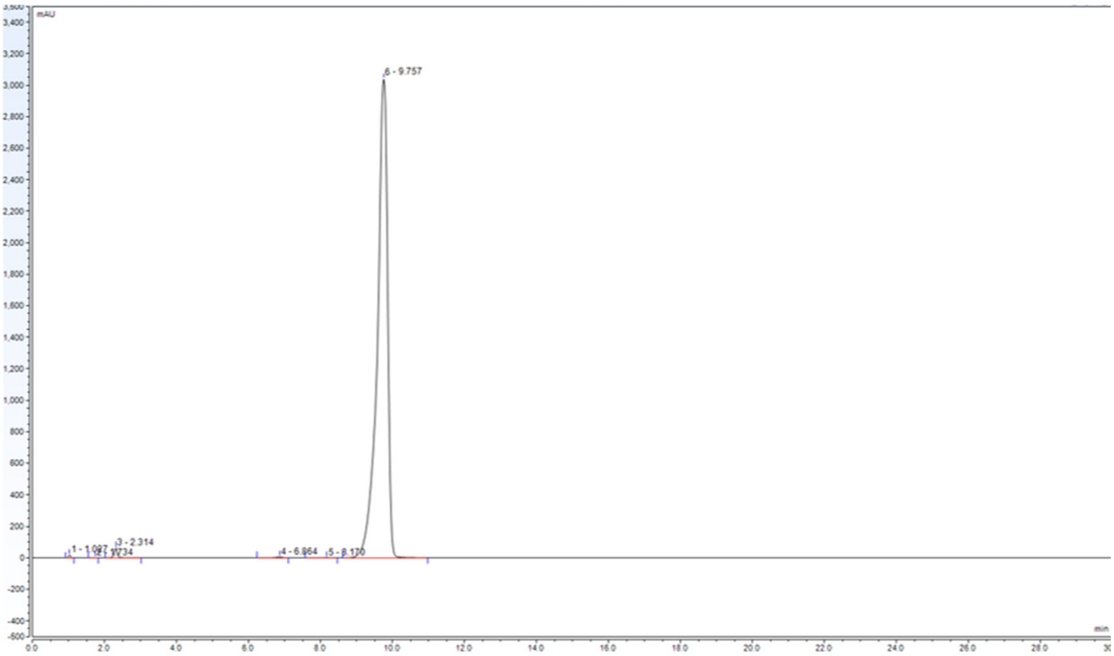

No.	Peak name	Retention Time<br>min	Relative Area<br>%	Area<br>mAU*min
1		1.027	0.11	1.0715
2		1.734	0.01	0.0853
3		2.314	0.62	6.2272
4		6.864	0.11	1.134
5		8.17	0.03	0.2979
6		9.757	99.12	998.6678

Figure S8 HPLC of compound **6**

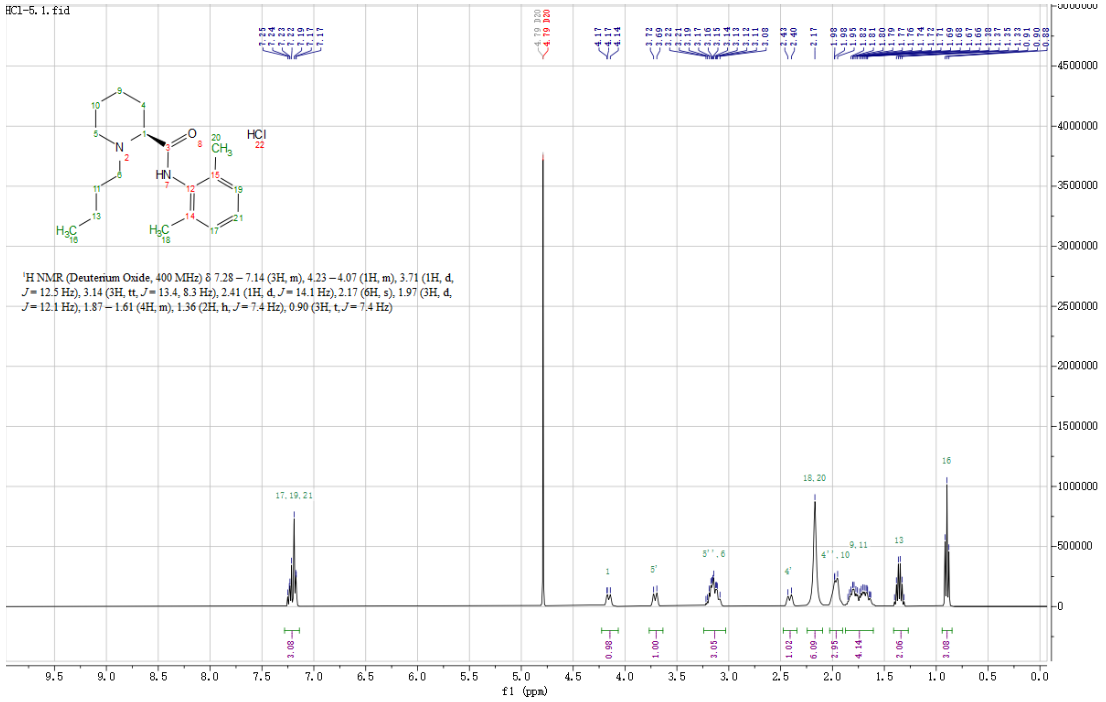

Figure S9 ^{1}H NMR of compound **21** (D_2O)

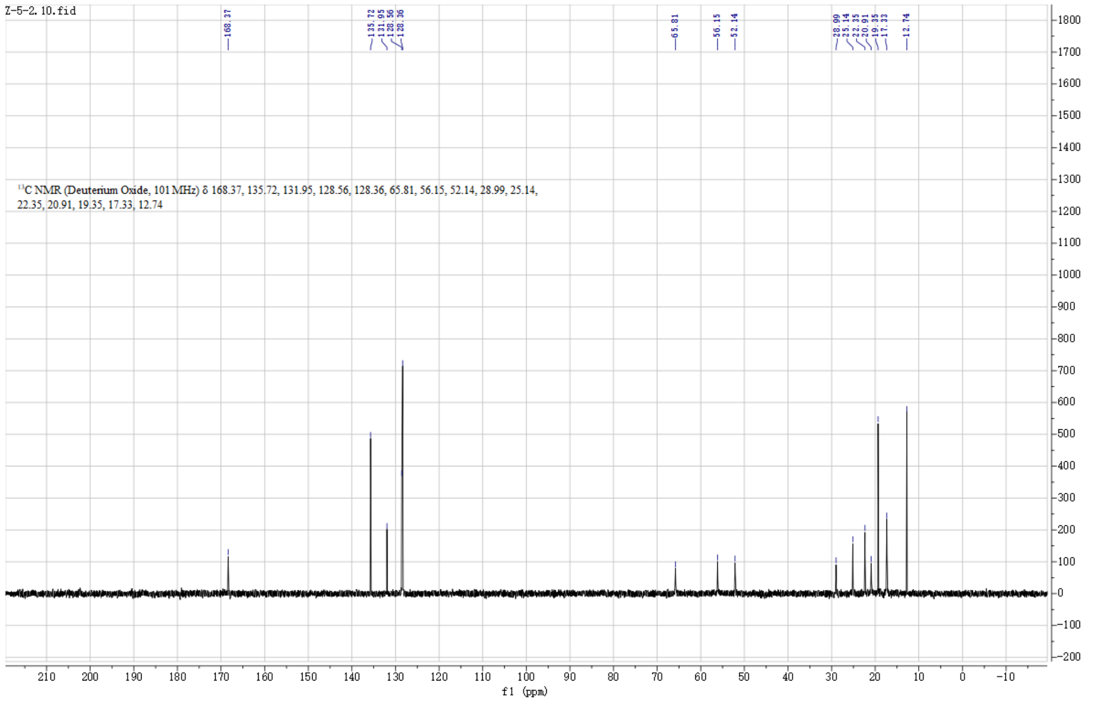

Figure S10 ^{13}C NMR of compound **21** (D_2O)

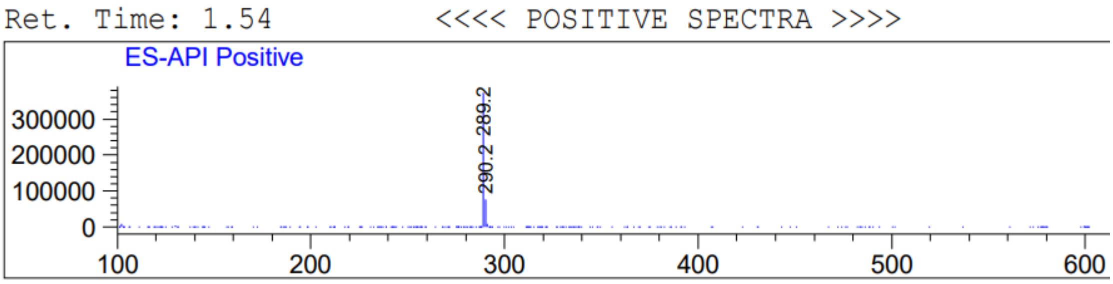

Figure S11 LC-MS of compound **21** (MW-HCl =288.2)

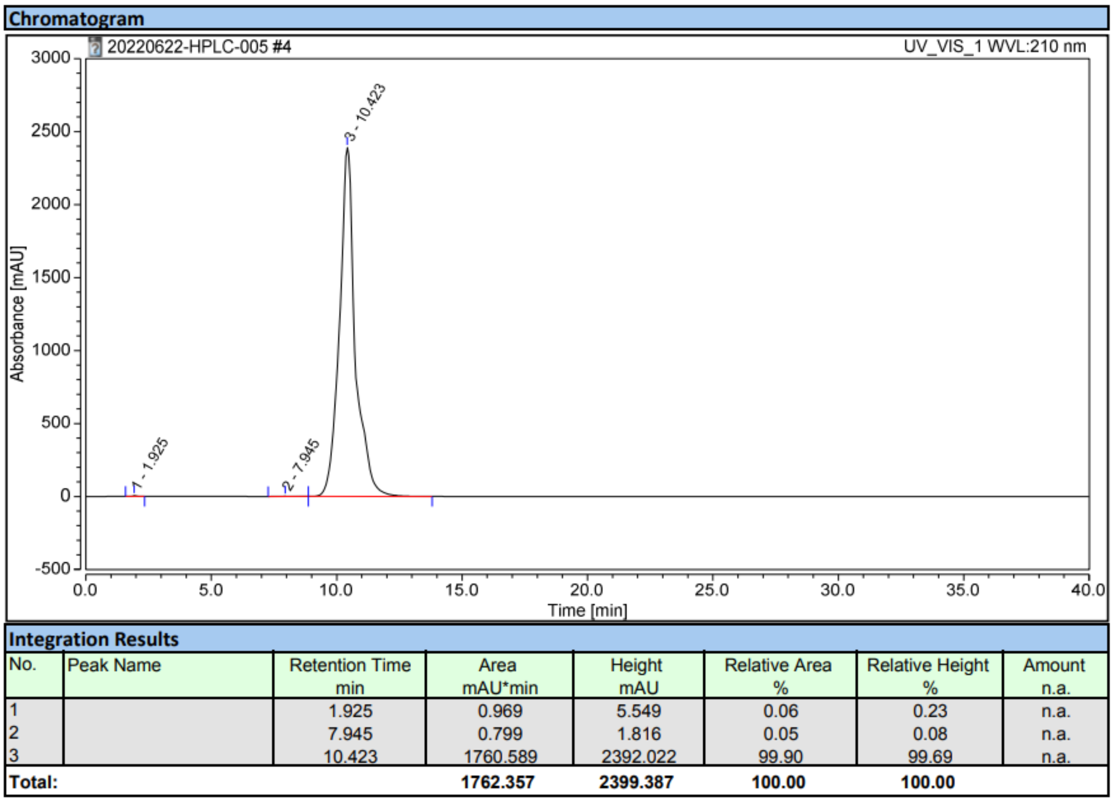

Integration Results

No.	Peak Name	Retention Time min	Area mAU*min	Height mAU	Relative Area %	Relative Height %	Amount n.a.
1		1.925	0.969	5.549	0.06	0.23	n.a.
2		7.945	0.799	1.816	0.05	0.08	n.a.
3		10.423	1760.589	2392.022	99.90	99.69	n.a.
**Total:**			**1762.357**	**2399.387**	**100.00**	**100.00**	

Figure S12 HPLC of compound **21** (chemically pure)

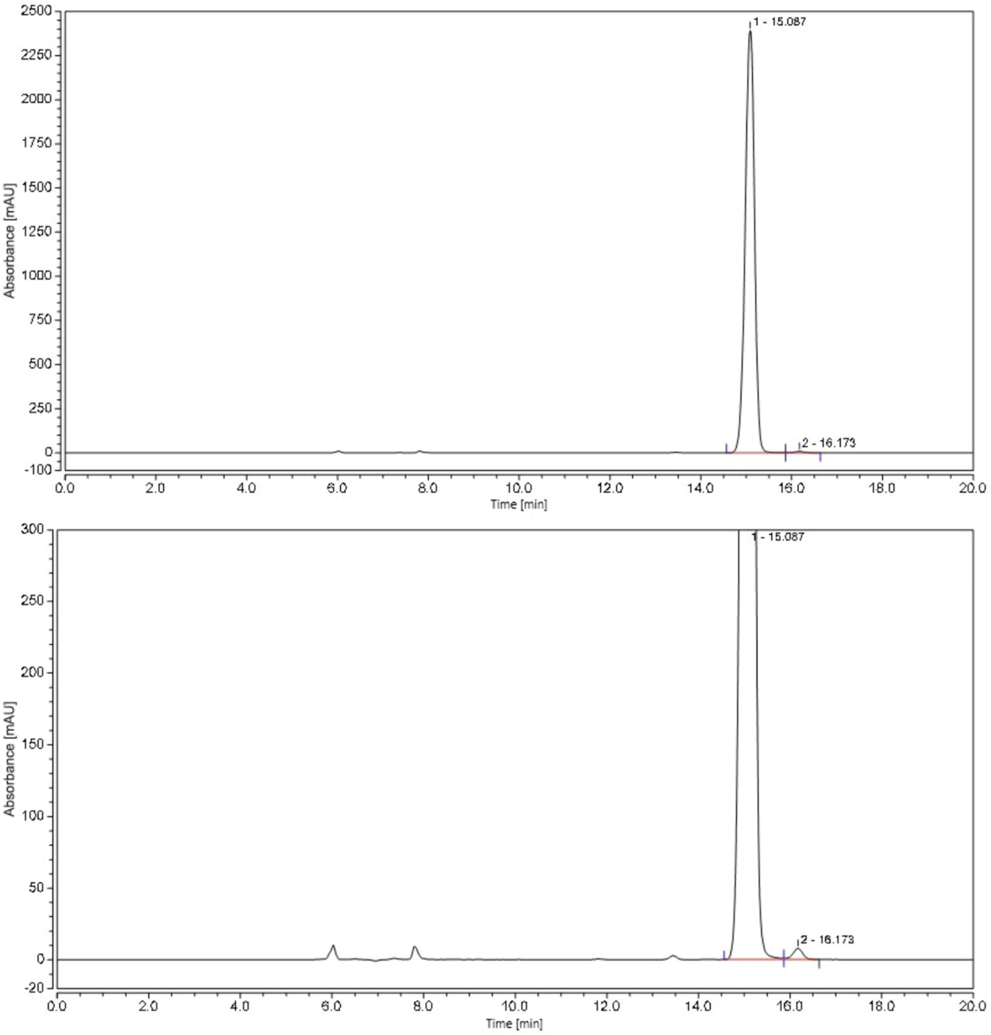

No.	Peak name	Retention Time min	Relative Area %	Area mAU*min	Resolution
1	levobupivacaine hydrochloride	15.087	99.65	569.9299	2.93
2	dextrobupivacaine hydrochloride	16.173	0.35	2.0292	n.a.

Figure S13 HPLC of compound **21** (optical pure)
